# Trends in self-reported psychological distress among college and university students from 2010 to 2018

**DOI:** 10.1017/S0033291719003350

**Published:** 2021-02

**Authors:** Marit Knapstad, Børge Sivertsen, Ann Kristin Knudsen, Otto Robert Frans Smith, Leif Edvard Aarø, Kari Jussie Lønning, Jens Christoffer Skogen

**Affiliations:** 1 Department of Health Promotion, Norwegian Institute of Public Health, Bergen, Norway; 2 Department of Psychosocial Science, Faculty of Psychology, University of Bergen, Bergen, Norway; 3 Department of Research & Innovation, Helse Fonna HF, Haugesund, Norway; 4 Department of Mental Health, Norwegian University of Science and Technology, Trondheim, Norway; 5 Centre for Disease Burden, Norwegian Institute of Public Health, Bergen, Norway; 6 Department of Psychosocial Science, University of Bergen, Bergen, Norway; 7 The Norwegian Medical Association, Oslo, Norway; 8 The Student Welfare Association of Oslo and Akershus (SiO), Oslo, Norway; 9 Alcohol and Drug Research Western Norway (KoRFor), Stavanger University Hospital, Stavanger, Norway; 10 Faculty of Health Sciences, University of Stavanger, Stavanger, Norway

**Keywords:** HSCL-25, anxiety, college students, depression, psychological distress, trend, university students

## Abstract

**Background:**

An increase in reported psychological distress, particularly among adolescent girls, is observed across a range of countries. Whether a similar trend exists among students in higher education remains unknown. The aim of the current study was to describe trends in self-reported psychological distress among Norwegian college and university students from 2010 to 2018.

**Methods:**

We employed data from the Students' Health and Wellbeing Study (SHoT), a nationwide survey for higher education in Norway including full-time students aged 18–34. Numbers of participants (participation rates) were *n* = 6065 (23%) in 2010, *n* = 13 663 (29%) in 2014 and *n* = 49 321 (31%) in 2018. Psychological distress was measured using the Hopkins Symptom Checklist-25 (HSCL-25).

**Results:**

Overall, a statistically significant increase in self-reported psychological distress was observed over time across gender and age-groups. HSCL-25 scores were markedly higher for women than for men at all time-points. Effect-size of the mean change was also stronger for women (time-by-gender interaction: χ^2^ = 70.02, df = 2, *p* < 0.001): in women, mean HSCL-25 score increased from 1.62 in 2010 to 1.82 in 2018, yielding a mean change effect-size of 0.40. The corresponding change in men was from 1.42 in 2010 to 1.53 in 2018, giving an effect-size of 0.26.

**Conclusions:**

Both the level and increase in self-reported psychological distress among Norwegian students in higher education are potentially worrying. Several mechanisms may contribute to the observed trend, including changes in response style and actual increase in distress. The relative low response rates in SHoT warrant caution when interpreting and generalising the findings.

## Introduction

The mental health of college and university students is a concern at campuses (Gallagher, 2015), as recently highlighted in the mass media (e.g. The Guardian, 2019) and research literature alike (Eisenberg, Gollust, Golberstein, & Hefner, 2007; Eisenberg, Hunt, Speer, & Zivin, 2011; Twenge et al., 2010; Xiao et al., 2017). Indeed, mental health problems, both in terms of self-reported psychological distress and mental disorders as assessed by clinical interviews, are highly prevalent in higher education student populations according to recent reports. Data from the World Health Organization World Mental Health Surveys across 21 countries indicated that one in five of college students had a 12-month DSM-IV disorder, as diagnosed using the WHO Composite International Diagnostic Interview (CIDI) (Auerbach et al., 2016). Another cross-national WHO survey, using web-based self-report questionnaires, showed that as many as one-third of first-year college students reported at least one DSM-IV anxiety, mood or substance disorder (Auerbach et al., 2018). Although overall prevalence estimates from diagnostic interviews seem to be similar for students and their non-student peers (Blanco, Okuda, Wright et al., 2008; Hunt & Eisenberg, 2010), the evidence regarding psychological distress is more mixed (Bernhardsdottir & Vilhjalmsson, 2013; Cvetkovski, Jorm, & Mackinnon, 2019), and some have found higher levels of psychological distress among students (Stallman, 2010). Consequences of psychological distress for function is less studied among students than in working age-populations, whereof impaired work function and capacity is found among workers at both clinical (Burton, Pransky, Conti, Chen, & Edington, 2004; Moussavi et al., 2007) and subthreshold symptom levels (Plaisier et al., 2010; Rai, Skapinakis, Wiles, Lewis, & Araya, 2010). Similar impact is, however, indicated among students across several domains (Alonso et al., 2018), such as less engagement in campus activities, poorer social and personal relations, lower academic performance (Keyes et al., 2012; Salzer, 2012), higher drop-out rates (Kessler, Foster, Saunders, & Stang, 1995; Mojtabai et al., 2015), as well as suicidal thoughts and behaviour (Keyes et al., 2012; Mortier et al., 2018).

Considering severe negative outcomes, it is worrying that an increase in psychological distress is observed among adolescents in several countries during the last few decades. The increase seems to be particularly evident for internalising problems among girls (Bor, Dean, Najman, & Hayatbakhsh, 2014; Collishaw, 2015). In Norway, the increase among adolescent girls is observed in terms of both self-reported psychological distress (Reneflot et al., 2018; Samdal et al., 2016), and as officially registered P codes within the International Classification of Primary Care (ICPC) system (including both psychological distress and mental disorders), proportions diagnosed with a mental disorder in specialist health care, and prescriptions of psychotropic medicine (Reneflot et al., 2018). Very little is known, however, with regards to mental health trends among college and university students. Data from a US survey showed that proportions of college students diagnosed or treated for mental disorders within the last 12 months had increased for most conditions from 2009 to 2015 (Oswalt et al., 2018). A similar increase in students with mental health problems is reported by US college counselling centres (Gallagher, 2015). Although the results of these studies of indirect mental health indicators point to increasing mental health problems among college and university students, studies reporting on trends in self-reported psychological distress are lacking.

The objective of the current study is therefore to describe trends in self-reported psychological distress, as measured by the Hopkins Symptom Checklist-25 (HSCL-25), among samples of Norwegian college and university students using three waves of a national health study of higher education conducted in 2010, 2014 and 2018. Due to the observed gender differences in trends in self-reported psychological distress found in adolescent populations, we also aimed to examine gender-by-time interaction effects.

## Methods

### Samples and procedure

The *SHoT* study (*Students*' *Health and Wellbeing Study*) is a national survey among students taking higher education in Norway, initiated by the three largest welfare organisations [*Sammen* (Bergen and surrounding area), *SiT* (Trondheim and surrounding area) and *SiO* (Oslo and Akershus)]. So far, three rounds of the survey have been conducted (2010, 2014 and 2018). The size and scope of the survey have expanded over time, but do in general cover mental and physical health and wellbeing, health-related behaviour, study-related information and demographics. Web-based platforms and email invitations with two reminders were used in all three waves. In the 2018 wave, SMS-invitation and one SMS reminder as well as an iPhone lottery was added, aiming to improve the response rate. Further details about SHoT study are described in a previous publication (Sivertsen, Råkil, Munkvik, & Lønning, 2019).

*The SHoT2010* targeted Norwegian full-time students <35 years of age in the three largest (Oslo, Bergen, Trondheim) and four smaller welfare organisations. It was conducted during the period from 11 October to 8 November 2010. A random sample (*n* = 26 779) was invited and *n* = 6053 students completed the survey, yielding a response rate of 22.6%.

*The SHoT2014* study targeted Norwegian full-time students <35 years of age in the 10 largest student welfare organisations in Norway. The data were collected in the period from 24 February 2014 to 27 March 2014. A random sample of *n* = 47 514, stratified by study institutions, faculties and departments, was invited, and *n* = 13 663 (28.5%) participated.

*The SHoT2018* study was a joint effort between the Norwegian Institute of Public Health (NIPH), the three largest welfare organisations (responsible board) and all student welfare organisations in Norway. All Norwegian full-time students, ⩽35 years of age taking higher education (both in Norway and abroad) were invited (*N* = 162 512). The data collection was conducted between 6 February and 5 April 2018. In total, 50 054 students completed the questionnaires, yielding a response rate of 30.8%. In the current study we included those aged <35 years, and excluded those aged 35 or more (*n* = 733), to align with the SHoT 2010 and 2014 samples, giving a final sample of *n* = 49 321.

### Ethics

Approvals for conducting the SHoT2010 and SHoT2014 studies were granted by the Data Protection Officer for research at the Norwegian Centre for Research Data. The SHoT2018 study was approved by the Regional Committee for Medical and Health Research Ethics in Western Norway (no. 2017/1176). Electronic informed consent was obtained after complete description of the study to the participants.

### Measurements

#### Self-reported psychological distress: Hopkins Symptom Checklist-25 (HSCL-25)

The Norwegian translation of HSCL-25 was used to measure psychological distress in all three surveys. This self-report measure is based on a longer checklist developed by Derogatis and colleagues (Derogatis, Lipman, Rickels, Uhlenhuth, & Covi, 1974). The scale consists of 25 statements regarding anxiety (10 items) and depressive (15 items) symptoms as experienced during the past 2 weeks, with response categories ‘not at all’ (1) to ‘extremely’ (4). Mean scores (1–4) were calculated, where a higher score indicated higher levels of psychological distress. The distribution of mean scores were somewhat right-skewed in all three waves (skewness: 2010 = 1.4; 2014 = 1.1; 2018 = 0.9, kurtosis: 2010 = 5.0; 2014 = 4.1; 2018 = 3.4, all *p* < 0.001). Several factor structures and cut-offs for clinical levels are proposed for the HSCL-25 (Glaesmer et al., 2014; Ventevogel et al., 2007). An investigation of the factor structure based on the SHoT 2014 data supported a uni-dimensional model in the student population (Skogen, Øverland, Smith, & Aarø, 2017). We have chosen to follow this recommendation in the current study, as similar structures presumably are valid also for the SHoT 2010 and SHoT 2018 data. Regarding clinical cut-offs, an average score of 1.75 was proposed in the original version (Winokur, Winokur, Rickels, & Cox, 1984) and widely used and recommended in several studies (Nettelbladt, Hansson, Stefansson, Borgquist, & Nordström, 1993; Veijola et al., 2003). Notably, other optimal cut-offs, i.e. best balancing specificity and sensitivity, and gender specific cut-offs have been suggested (e.g. Sandanger et al., 1998). In addition, most validation studies were conducted several years ago and the optimal cut-off for student populations is to the best of our knowledge not examined. In descriptive reports from the SHoT surveys (Knapstad, Heradstveit, & Sivertsen, 2018; Nedregård & Olsen, 2011, 2014), a cut-off at ⩾2.0 have been employed in addition to the ⩾1.75 cut-off. This practice has been based on experiences from the welfare organisations that relatively high symptoms loads are common among students. In the current study, we have chosen to align with this practice. Due to the uncertainty concerning optimal cut-off and their implication (as elaborated on in the ‘Discussion’ section), however, relatively more attention was given to changes in continuous scores rather than the dichotomised.

#### Demographic characteristics

The following demographic characteristics were self-reported in all three surveys, and included in order to compare sample characteristics between the surveys on variables that may be related to the level of psychological distress: sex (male, female), age (coded as 18–20 years, 21–22 years, 23–25 years and 26–34 years), household status (coded as ‘living alone’ *v.* ‘living with others’) and relationship status (coded as ‘single’ *v.* ‘married’/‘partner’ or ‘girl-/boy-friend’).

### Statistical analyses

In order to assess the comparability of responses across the three surveys, we investigated the overall factor structure of HSCL-25. Informed by previous findings from the SHoT 2014 study and using confirmatory factor analysis (CFA) we estimated the fit of a 1-factor model. Overall, the 1-factor model yielded a satisfactory fit (RMSEA = 0.077, CFI = 0.938, TLI = 0.932). Next, we investigated if this 1-factor model was configural (equivalent model form) and scalar invariant (equivalent item thresholds) across the survey years. The model was considered measurement non-invariant if the change in model fit was more than 0.015 for RMSEA and more than −0.010 for CFI (Putnick & Bornstein, 2016). All CFA analyses were performed using variance-adjusted weighted least squares estimators suitable for ordinally scaled responses. Based on these analyses, the HSCL-25 was found both configural invariant (*v.* overall: ΔRMSEA < 0.001, ΔCFI = 0.002) and scalar invariant (*v.* configural: ΔRMSEA = −0.015, ΔCFI = 0.014) across time.

Due to the commonly observed gender difference in psychological distress and studies indicating a steeper increase in psychological distress among teenage girls compared to boys (Bor et al., 2014; Collishaw, 2015; Reneflot et al., 2018), evidence of an interaction effect between gender and survey year was examined comparing regression models with and without the interaction term using the likelihood-ratio test (LRT). The LRT gave support of such interaction effect (χ^2^ = 70.02, df = 2, *p* < 0.001). Thus, we chose to run all further analyses stratified by gender.

Trends in self-reported psychological distress were initially examined by comparing changes in the total HSCL-25 score [mean (95% confidence interval (CI))] as well as proportions (95% CI) scoring above the cut-offs across the three surveys. We conducted linear regression (for means) and logistic regression (for proportions above cut-off) models to determine significant differences between the study years. Test for trend was done by treating the predictor (study year) as a linear variable in the regression models. Effect sizes of change in mean scores were calculated using Cohen's *d* for independent samples (mean difference/pooled standard deviation by gender and across pairs of years) between 2010 and 2018 and between 2014 and 2018, respectively. Finally, as a sensitivity analysis, we examined change in score per single HSCL-25 item to explore to what degree the direction and strength of change in mean scores were consistent across items and whether the CIs were overlapping.

As the three surveys included somewhat different welfare organisations and institutions, sensitivity analyses were also performed comprising the institutions included in all three surveys only. The sub-samples included in these analyses were *n* = 4369 (72.2% of total sample) in 2010, *n* = 6681 (48.9%) in 2014 and *n* = 24 298 (49.2%) in 2018. These analyses were performed using the same statistical tests as for the main analyses.

CFA and the measurement invariant analyses were performed in Mplus 8 (Muthén & Muthén, 1998–2017) and all other analyses were performed using Stata 15.0 (StataCorp, 2017).

## Results

### Demographic characteristics of survey participants in 2010, 2014 and 2018

Table 1 details the gender and age distribution of the survey participants in 2010, 2014 and 2018. Women constituted around two of three participants in all three surveys, with a slight increase in proportion of women from 2010 to 2018 (χ^2^ = 52.6, df = 2, *p* < 0.001). This differs slightly from the gender distribution in higher education in Norway during the same period (around 60% women) (Statistics Norway, 2018). All three datasets included both younger and older students, though there were fewer students in the youngest age-group in the 2014 survey compared to the 2010 and 2018 surveys.

**Table 1.** Descriptive statistics of study participants in the three SHoT waves

	2010		2014		2018		
	*n*	%	*n*	%	*n*	%	Difference
	6053		13 663		49 321		
Gender							χ^2^ = 52.6, df = 2, *p* < 0.001
Women	3982	65.79	9082	66.47	33 927	69.08	
Men	2071	34.21	4581	33.53	15 184	30.92	
Age-groups							χ^2^ = 620.8, df = 6, *p* < 0.001
18–20	1237	20.44	1767	12.93	8832	18.17	
21–22	1711	28.27	3678	26.92	15 471	31.83	
23–25	1921	31.74	4887	35.77	15 902	32.71	
26–34	1184	19.56	3331	24.38	8404	17.29	

### Trends in HSCL-25 score across survey years

**Fig. 1.**
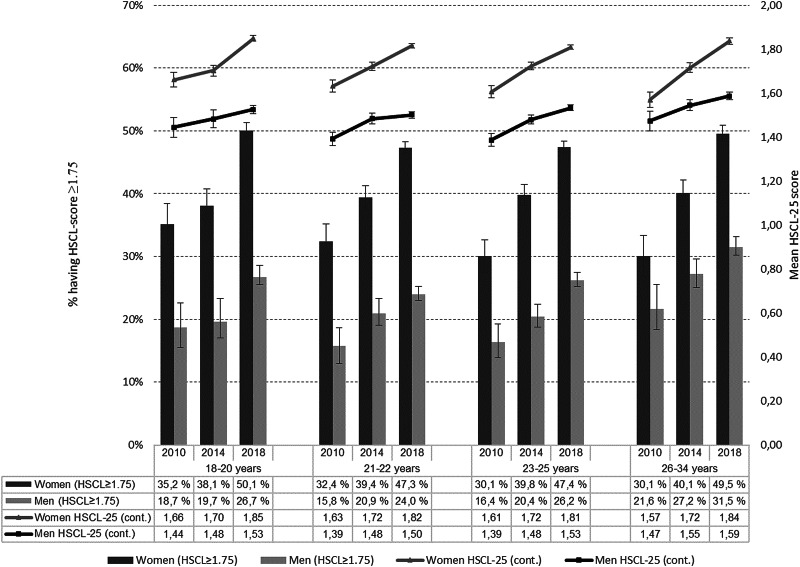
Mean (95% CI) HSCL-25 score and percentages (95% CI) scoring ⩾1.75 per survey year, stratified by gender and age-group.

Overall, an increase in self-reported psychological distress was observed between each time point from 2010 to 2018 (treating time as a categorical variable) and as an overall trend (treating time as continuous variable) (all *p* < 0.001). The increase was found when using both continuous and dichotomous HSCL-25 scores, for both female and male students and in all age-groups (Fig. 1 and Table 2).

**Table 2.** Trends in mean (95% CI) HSCL-25 scores and percentages (95% CI) scoring ⩾1.75 and ⩾2.0 from 2010 to 2018, gender-stratified

	2010		2014		2018			
	Mean	95% CI	Mean	95% CI	Mean	95% CI	Trend^a^	Effect size
Women	1.62	1.60–1.63	1.72	1.71–1.73	1.82	1.82–1.83	2010 < 2018***	0.40
							2014 < 2018***	0.20
							2010 < 2014***	0.21
Men	1.42	1.40–1.43	1.50	1.49–1.51	1.53	1.53–1.54	2010 < 2018***	0.26
							2014 < 2018***	0.08
							2010 < 2014***	0.19
	%	95% CI	%	95% CI	%	95% CI		
Women							2010 < 2018***	
⩾1.75	31.8	30.4–33.3	39.5	38.5–40.5	48.3	47.8–48.8	2014 < 2018***	
⩾2.00	19.1	17.8–20.3	25.0	24.1–25.9	33.8	33.3–34.3	2010 < 2014***	
Men							2010 < 2018***	
⩾1.75	17.7	16.1–19.4	22.4	21.2–23.6	26.8	26.1–27.5	2014 < 2018***	
⩾2.00	9.5	8.3–10.8	13.4	12.4–14.4	16.9	16.3–17.5	2010 < 2014***	

a Differences in means examined by linear regression models. Differences in above cut-off scores examined by logistic regression models.

***p < 0.001.

Mean HSCL-25 scores where markedly higher for women than for men at all three time points and the effect size of the mean change was also higher for women than for men (Table 2): in women the mean HSCL-25 score increased gradually from 1.62 (1.60–1.63) in 2010 to 1.82 (1.82–1.83) in 2018 (*p* < 0.001), giving an effect size of mean change from 2010 to 2018 of 0.40. The corresponding change in mean score in men was from 1.42 (1.40–1.43) in 2010 to 1.53 (1.53–1.54) in 2018 (*p* < 0.001), yielding an effect size of 0.26.

As many as 48.3 (47.8–48.8)% of the female students and 26.8 (26.1–27.5)% of the male students participating in the 2018 survey scored above the ⩾1.75 cut-off. Employing the ⩾2.0 cut-off, the corresponding proportions were 33.8 (33.3–34.3)% for female and 16.9 (16.3–17.5)% for male students. Compared to the 2010 survey this constituted a 16.5 percentage point increase among the female students and a 9.1 percentage point increase among the male students scoring above the ⩾1.75 cut-off (Table 2).

### Sensitivity analyses

#### Individual items

We explored the degree of change for all individual HSCL-25 items, and found an increase in mean score with non-overlapping CIs from 2010 to 2018 for all except for two items (‘faintness, dizziness or weakness’ and ‘feeling restless, can't sit still’). For somatic anxiety symptoms among men, the CIs of mean scores were overlapping comparing 2014 and 2018. None of the items showed a decrease in score from 2010 to 2018 (data not shown).

#### Adjustments and same institutions across surveys

Adjusting for demographic factors (age-group, household status and relationship status) in regression analyses hardly changed the estimated association between HSCL-25 score and survey year (data not shown). Finally, including only the institutions included in all three surveys gave overall similar findings as for the main analyses. Also within these sub-samples evidence for a gender-by-time interaction effect was found (LR χ^2^ = 27.64, *p* < 0.001). The mean HSCL-25 scores in the sub-samples were slightly lower than in the main samples, but effect sizes of the mean change remained similar and all regression analyses were significant at a *p* < 0.001 level. For example, in women the HSCL score changed from 1.61 (s.d. = 0.48) in 2010 to 1.80 (s.d. = 0.55) in 2018, ES = 0.37, and in men from 1.41 (s.d. = 0.42) to 1.52 (s.d. = 0.47), ES = 0.26.

## Discussion

### Main findings

The objective of the current study was to provide empirical evidence regarding trends in self-reported psychological distress among students in higher education. Employing data from three waves of a large, national survey among Norwegian full-time college and university students, a clear increase in self-reported psychological distress was observed from 2010 to 2018 across gender and age-groups. The increase was more pronounced among the female than among the male students, as indicated by moderate effect size (ES = 0.40) for the change in mean HSCL-25 score from 2010 to 2018 among women and small effect size (ES = 0.26) of change among men during the same period. The increase was furthermore observed across almost all single HSCL-25 items and could not be explained by factors such as age, relationship status and household status or selection of institutions.

### Interpretation of findings

The observed increase in self-reported psychological distress aligns with the bulk of studies in adolescent populations, both Norwegian (Reneflot et al., 2018; Samdal et al., 2016; Statistics Norway, 2017) and from other Western countries (Bor et al., 2014; Collishaw, 2015). Similar to the current study, the majority of these show a steeper or more pronounced increase in psychological distress among girls than among boys. The time periods examined in previous studies varies, and few directly overlap with the period covered in the current study. Furthermore, students in higher education are somewhat older and in a different life situation. Thus, there might be other time trends, in general or specifically for the higher education student population, impacting on the current findings than the abovementioned. We are not aware of other trend studies among college or university students having direct measures of self-reported psychological distress. Our findings should nonetheless be seen in relation to the findings from the study among US college students (Oswalt et al., 2018), reporting an increase of self-reported diagnoses and use of mental health services during a time period overlapping (2009–2015) with the current data.

Also when applying conventional cut-off scores, a clear increase in proportions with high symptomatology was observed, both within the current study and when comparing current results to that of previous studies in related populations. For instance, a survey by Statistics Norway applying HSCL-25 found 6% and 14% of men/women aged 16–24 to score above the ⩾1.75 cut-off in 1998 and 11% and 25% to do so in 2012 (Statistics Norway, 2017). A study of Norwegian undergraduate students from 2006 (Nerdrum, Rustøen, & Rønnestad, 2006), found that 21% reported ‘clinical significant psychological distress’ (using the General Health Questionnaire). In the SHoT 2018 as many as 27% of men and 48% of women had a mean score ⩾1.75. Such high estimates are disturbing, and urges indeed further monitoring and examination of generalisability to the whole student population. The numbers should, however, not be interpreted in clinical terms (Thombs, Kwakkenbos, Levis, & Benedetti, 2018). First, no validation studies of the HSCL-25 against structural clinical interviews have been performed among students in higher education. This is in particular relevant as younger adults typically show higher symptom loads than older (Westerhof & Keyes, 2010). Second, existing validation studies were mostly conducted during the 1990s and have found rather weak correspondence between a high HSCL-25 score and clinical diagnoses, with relatively low specificity and sensitivity (Batterham, Sunderland, Slade, Calear, & Carragher, 2018; Mattisson, Bogren, & Horstmann, 2013; Sandanger et al., 1998; Veijola et al., 2003). For example, based on a sensitivity of 76% and a specificity of 73% (Nettelbladt et al., 1993) for a ⩾1.75 cut-off, and assuming a true prevalence of 20%, 7515 are correctly identified as cases and 28 872 are correctly identified as non-cases in the SHoT 2018 sample. However, 10 679 are false positives, constituting 58.7% of those classified as cases. If the true prevalence is lower, the exaggeration of the prevalence will be even higher (Thombs et al., 2018). It has thus been suggested that a high HSCL-25 score should be seen as an indicator of psychosocial stress rather than a diagnostic condition (Mattisson et al., 2013; Sandanger et al., 1999). There is also a discussion of whether the ‘everyday language’ employed in HSCL-25 may probe recognition and normalisation of psychological distress (Sandanger et al., 1999). Alternatively, one could argue that this may contribute giving normal conditions clinical labels. Relatedly, an increase in psychological distress does not necessarily indicate a similar increase in mental disorders. There is no evidence of an increase in the latter in the general population (Baxter et al., 2014). It could be that the former is more sensitive for societal changes, such as changes in lifestyle factors and ways of perceiving and handling mental health problems, while the latter is more stable and to a greater extent influenced by genes, biology and more severe life events (Baxter et al., 2014). More research is needed to get a better understanding of similarities and differences in the risk factors and eliciting factors between subclinical and clinical mental conditions. Also, both quantitative and qualitative studies are needed to examine the causes, meaning and implications of psychological distress among those showing elevated HSCL-25 scores.

#### Possible causes of the observed increase in self-reported psychological distress

Several causes are possible of the observed increase, of which broadly may sort under two main categories; whether it is true and if so, what the increase can be attributed to. Regarding the first, the relatively modest participation rates with little information about non-participants, makes it difficult to fully evaluate to what extent the participants in the current study were representative of the general student population. Selection bias could have affected the observed trend to the extent that the probability to participate conditional on mental health status varied by survey. For example, reduced stigma may have increased the participation rate of those with mental health problems between 2010 and 2018. In addition, variations in recruitment procedures and questionnaire setup between the SHoT surveys may also have contributed to differential selection bias across surveys. It can, for example, not be precluded that the IPhone lottery that was added in 2018 may have increased the participation rate of those with low material affluence, a variable robustly associated with mental health problems. In contrast, those participating after reminders were sent out unexpectedly had somewhat lower HSCL-25 scores than those responding before the reminders (*d* < 0.2) (Knapstad et al., 2018). All in all, it is unclear whether and to what extent differential selection bias explains the observed trend. Finally, though all surveys were marketed to concern students' health and wellbeing (positively and negatively), student newspapers pre the 2018 wave often focused on the poor mental health observed in previous waves. Thus, one may speculate whether some participants have associated the SHoT 2018 with this, and that their responses were correspondingly ‘primed’ towards reporting more symptoms (Goodwin et al., 2013).

If one assumes the trend is (partly) true, the next important challenge is to identify conditions and factors that may contribute to explaining the observed increase in reported levels of distress. Some explanations could be suggested. First, an increasing proportion of the Norwegian youth population pursue higher education. Consequently, the student population is increasingly heterogeneous and might include a larger amount of individuals having fewer resources to cope with the demanding student life situation and/or having mental health problems in general. Over the relative short time period from 2010 to 2018 this is, however, not likely to explain a substantial part of the observed increase in psychological distress [between 2007 and 2017 the proportion of Norwegian 19–24 year olds taking higher education only rose from 30% to 35% (Statistics Norway, 2018)].

Second, a central discussion concerns whether the observed increase reflects more focus on and openness regarding mental health problems rather than changes in experiences of distress *per se* (Baxter et al., 2014; Collishaw, 2015; Much & Swanson, 2010). That is, whether these factors have improved recognition of and/or lowered the threshold to report mental health problems. Related, there might be changes in ways of perceiving or expressing problems or threshold for evaluating symptoms as problematic. Some attempts to test the ‘openness’ hypothesis have been carried out in adolescent populations, e.g. by comparing changes in positive and negative framed items and by examining whether changes have been restricted to some types of symptoms or conditions. Thus far, findings have been mixed or not yielded support for the hypothesis (Collishaw, 2015; von Soest & Wichstrøm, 2014). The possibility is difficult fully to preclude, however, and the few existing studies have often been hampered by weaknesses in designs, such as non-equivalent measures over time or differences between samples included (Sweeting, West, Young, & Der, 2010; Sweeting, Young, & West, 2009). In the current study, the increase was evident across almost all items examined, which might lend support to an increased openness. Interestingly, the single items *not* increasing from 2014 to 2018 in men all regarded somatic symptoms, rather than emotional and cognitive. One might thus speculate whether this mirrors a change in how to handle symptoms more than increased distress *per se* in men. To explain the observed gender difference in increase, however, women need to have been more responsive of the increased attention towards mental health issues and/or towards openness. Also, the confirmatory factor analyses indicated that HSCL-25 is a uni-dimensional scale, and does not distinguish well between aspects of mental health, such as anxiety, depression and psychosomatic symptoms (Skogen et al., 2017).

Finally, several risk factors have been suggested to contribute to an actual deterioration of the mental health of youth and students, such as an increase in perfectionism (Curran & Hill, 2017), individualism and focus on appearance (Twenge et al., 2010; von Soest & Wichstrøm, 2014), educational expectations (Sweeting et al., 2010; West & Sweeting, 2003), negative impact of social media, and changes in drug use, in particular cannabis use (Baxter et al., 2014; Thombs et al., 2018; von Soest & Wichstrøm, 2014). It has also been discussed whether there are changes in vulnerability to risk factors and the extent rising generations hold mastery skills of the normal stress and strains of life. There is thus far little existing evidence in support of an increased vulnerability (Sweeting et al., 2010; von Soest & Wichstrøm, 2014), except for possibly increased worry about school and among females worry about family relationships (Sweeting et al., 2010), and none have examined this in higher education populations. In the Norwegian student context, there are for instance discussions on whether the upper limit of loans from the Norwegian State Educational Loan Fund is insufficient to make a living, putting an increased pressure on students to work alongside their studies. Notably, though, in SHoT the proportions reporting difficulties in coping with the running costs have remained quite stable from the 2010 to the 2018 survey (Knapstad et al., 2018). As for the potential impact of increased openness, it is difficult to establish causal links or to point out single factors contributing to the observed deterioration of mental health among young adults. The matrix of risk and protective factors contributing to mental health and mental health problems is complex and multidimensional, constituting both proximal and distal factors interacting over time and across structures. If the observed increase reflects a real change in distress, there is reason to believe that several different risk factors are relevant. These include both risk factors which are changing over time (exposure) and stable risk factors, as also changing relationships between risk factors can impact mental health (through interaction and vulnerability) (Collishaw, 2015; von Soest & Wichstrøm, 2014). Future studies should investigate whether changes in levels of distress could be explained by factors and clusters of factors such as those mentioned above.

### Strengths and limitations

The most important limitation of the current study was the relative modest participation rates in all three surveys (23%, 29% and 31%). The possible impact of selection bias on the observed trend is discussed above. Poor mental health is in general found related to non-participation, and would as such lead to an underestimation of the true level of psychological distress among students (Knudsen, Hotopf, Skogen, Øverland, & Mykletun, 2010; Torvik, Rognmo, & Tambs, 2012). Selective participation could also bias the absolute frequencies observed to the extent the selection was correlated with the HSCL-25 score. We could not apply survey weights, as we did not have similar data regarding e.g. age and region available across the three waves. The 2010 and 2014 waves constituted random samples from a selection of educational institutions only, and we do unfortunately not have exact data on the age distribution of the frame population for these samples. The sampling frames for the regions are also different between the waves. The uncertainty regarding the cut-offs of the HSCL-25, as also discussed above, makes it uncertain to what extent the observed scores translate into prevalence estimates of mental disorders and we do not know the functional impact of the distress. Thus, the results cannot be used as an indicator of mental health service needs in the student population. The lack of a non-student comparison group precluded examination of whether/to what extent a similar trend is evident among young people in Norway in general.

The main strengths of the study include the large sample sizes, in 2018 inviting all full-time Norwegian college and university students, the use of the same, well-acknowledged instrument of psychological distress in all three surveys, and the investigation and confirmation of measurement invariance of HSCL-25 across surveys.

### Implications for public health and research

College and university students represent a large and growing segment of the Norwegian population. Increase in psychological distress in this group may thus have enormous societal impact in addition to the consequences for the affected individuals, for instance taking into account the associated negative impact on educational attainment (Kessler et al., 1995; Mojtabai et al., 2015). As discussed, studies obtaining higher participation rates and/or including registry-linkage to illuminate the degree of systematic selection are needed to confirm whether the observed level and trend is representative of the student population as a whole. More knowledge is also needed about the lived experience and functional impairments associated with the levels of self-reported psychological distress among college and university students. Such investigations should look at potential risk factors, consequences and reduced functioning both in the short- and long-terms. Though an increase in help seeking is observed in some studies (Oswalt et al., 2018), there are several indications that still only a minority seek and receive adequate help for their mental health problems (Arria et al., 2011; Auerbach et al., 2016; Blanco et al., 2008). Thus, in addition to increase our understanding of the scope of psychological distress in this population, further research may also inform strategies in how to prevent or reduce distress, and how best to reach out to help students cope during their studies when needed. Finally, a continuous focus on preventive measures is obviously warranted by both universities and health authorities, to ensure health promoting and inclusive arenas for all students.
